# Supplementary material for: The interactome of intact mitochondria by cross-linking mass spectrometry provides evidence for coexisting respiratory supercomplexes
Source: Mol Cell Proteomics. 2017 Dec 8;17(2):216–32. doi: 10.1074/mcp.RA117.000470 (PMC5795388; doi:10.1074/mcp.RA117.000470)
Supplement: Supplemental Data [file supp_RA117.000470_133922_0_supp_23978_fzffwf.zip › spectra_annotation/mito_spectra_annotation/341-1-1-1-3-1.pdf]

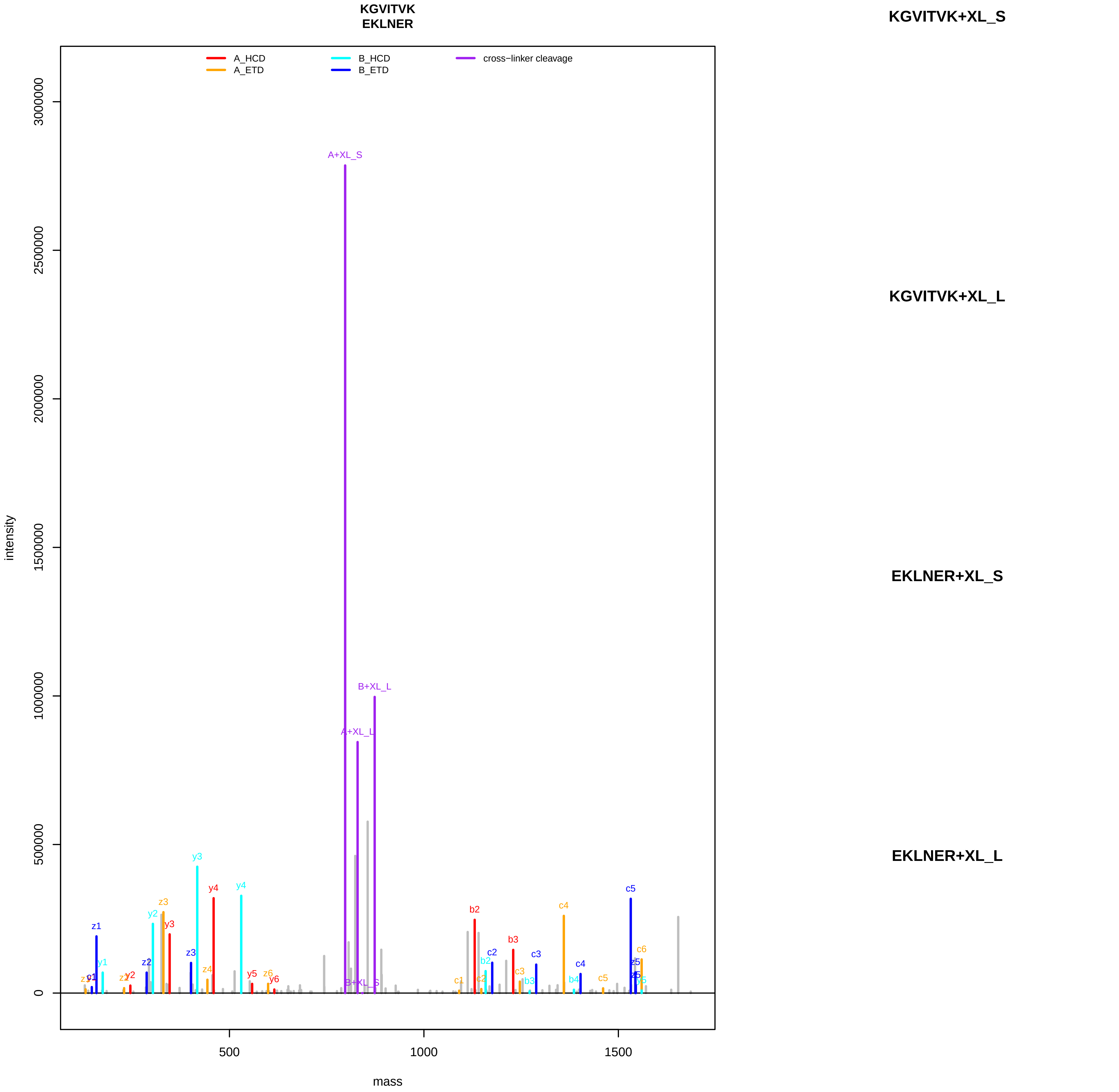
KGVITVK
EKLNER
KGVITVK+XL_S
KGVITVK+XL_L
EKLNER+XL_S
EKLNER+XL_L
A_HCD
A_ETD
B_HCD
B_ETD
cross−linker cleavage
intensity
mass
0
500000
1000000
1500000
2000000
2500000
3000000
500
1000
1500
A+XL_S
B+XL_L
A+XL_L
B+XL_S
y1
y2
y3
y4
y5
y6
z1
z2
z3
z4
z5
z6
c1
c2
c3
c4
c5
c6
b2
b3
b4
